# Definitions and guidelines for research on antibiotic persistence

**DOI:** 10.1038/s41579-019-0196-3

**Published:** 2019-04-12

**Authors:** Nathalie Q. Balaban, Sophie Helaine, Kim Lewis, Martin Ackermann, Bree Aldridge, Dan I. Andersson, Mark P. Brynildsen, Dirk Bumann, Andrew Camilli, James J. Collins, Christoph Dehio, Sarah Fortune, Jean-Marc Ghigo, Wolf-Dietrich Hardt, Alexander Harms, Matthias Heinemann, Deborah T. Hung, Urs Jenal, Bruce R. Levin, Jan Michiels, Gisela Storz, Man-Wah Tan, Tanel Tenson, Laurence Van Melderen, Annelies Zinkernagel

**Affiliations:** 1 Racah Institute of Physics, The Hebrew University, Jerusalem, Israel; 2 MRC Centre for Molecular Bacteriology and Infection, Imperial College London, London, UK; 3 Department of Biology, Northeastern University, Boston, MA USA; 4 Institute of Biogeochemistry and Pollutant Dynamics, ETH Zurich, Zurich, Switzerland; 5 Department of Environmental Microbiology, Eawag, Dubendorf, Switzerland; 6 Department of Molecular Biology and Microbiology, Tufts University School of Medicine, Boston, MA USA; 7 Department of Medical Biochemistry and Microbiology, Uppsala University, Uppsala, Sweden; 8 Department of Chemical and Biological Engineering, Princeton University, Princeton, NJ USA; 9 Focal Area Infection Biology, Biozentrum of the University of Basel, Basel, Switzerland; 10 Institute for Medical Engineering & Science, Department of Biological Engineering, and Synthetic Biology Center, Massachusetts Institute of Technology, Cambridge, MA USA; 11 Wyss Institute for Biologically Inspired Engineering, Harvard University, Boston, MA USA; 12 Broad Institute of MIT and Harvard, Cambridge, MA USA; 13 Department of Immunology and Infectious Diseases, Harvard T. H. Chan School of Public Health, Boston, MA USA; 14 Institut Pasteur, Genetics of Biofilms Laboratory, Paris, France; 15 Institute of Microbiology, ETH Zurich, Zurich, Switzerland; 16 Molecular Systems Biology, Groningen Biomolecular Sciences and Biotechnology Institute, University of Groningen, Groningen, Netherlands; 17 Department of Biology, Emory University, Atlanta, GA USA; 18 Center for Microbiology, KU Leuven–University of Leuven, Leuven, Belgium; 19 Division of Molecular and Cellular Biology, Eunice Kennedy Shriver National Institute of Child Health and Human Development, Bethesda, MD USA; 20 Infectious Diseases Department, Genentech, South San Francisco, CA USA; 21 Institute of Technology, University of Tartu, Tartu, Estonia; 22 Faculté des Sciences, Université Libre de Bruxelles, Bruxelles, Belgium; 23 Division of Infectious Diseases, University Hospital Zurich, University of Zurich, Zurich, Switzerland

**Keywords:** Antibiotics, Bacterial physiology, Antimicrobial resistance, Antibacterial drug resistance

## Abstract

Increasing concerns about the rising rates of antibiotic therapy failure and advances in single-cell analyses have inspired a surge of research into antibiotic persistence. Bacterial persister cells represent a subpopulation of cells that can survive intensive antibiotic treatment without being resistant. Several approaches have emerged to define and measure persistence, and it is now time to agree on the basic definition of persistence and its relation to the other mechanisms by which bacteria survive exposure to bactericidal antibiotic treatments, such as antibiotic resistance, heteroresistance or tolerance. In this Consensus Statement, we provide definitions of persistence phenomena, distinguish between triggered and spontaneous persistence and provide a guide to measuring persistence. Antibiotic persistence is not only an interesting example of non-genetic single-cell heterogeneity, it may also have a role in the failure of antibiotic treatments. Therefore, it is our hope that the guidelines outlined in this article will pave the way for better characterization of antibiotic persistence and for understanding its relevance to clinical outcomes.

## Abstract (web summary)

Antibiotic persistence contributes to the survival of bacteria during antibiotic treatment. In this Consensus Statement, scientists working on the response of bacteria to antibiotics define antibiotic persistence and provide practical guidance on how to study bacterial persisters.

## Introduction

More than 70 years ago, Hobby^1^ and Bigger^2^ observed that antibiotics that are considered bactericidal and kill bacteria in fact fail to sterilize cultures. Bigger realized that the small number of bacteria that manage to survive intensive antibiotic treatments are a distinct subpopulation of bacteria that he named ‘persisters’.

Fuelled in part by increasing concerns about antibiotic resistance but also by technological advances in single-cell analyses, the past 15 years have witnessed a great deal of research on antibiotic persistence by investigators with different backgrounds and perspectives. As the number of scientists that tackle the puzzles and challenges of antibiotic persistence from many different angles has profoundly increased, it is now time to agree on the basic definition of persistence and its distinction from the other mechanisms by which bacteria survive exposure to bactericidal antibiotic treatments^3^. Several approaches have independently emerged to define and measure persistence. Research groups following seemingly similar procedures may reach different results, and careful examination of the experimental procedures often reveals that results of different groups cannot be compared. During the European Molecular Biology Organization (EMBO) Workshop ‘Bacterial Persistence and Antimicrobial Therapy’ (10–14 June 2018) in Ascona, Switzerland, which brought together 121 investigators involved in antibiotic persistence research from 21 countries, a discussion panel laid the main themes for a Consensus Statement on the definition and detection procedure of antibiotic persistence detailed below. In light of the potential role that antibiotic persistence can have in antibiotic treatment regimens, it is our hope that clarification and standardization of experimental procedures will facilitate the translation of basic science research into practical guidelines.

## Defining the persistence phenomena

We adopt here a phenomenological definition of antibiotic persistence that is based on a small set of observations that can be made from experiments performed in vitro and that does not assume a specific mechanism. We focus on the differences and similarities between antibiotic persistence and other processes enabling bacteria to survive exposure to antibiotic treatments that could kill them, such as resistance, tolerance and heteroresistance. We identify different types of persistence that should be measured differently to obtain meaningful results; therefore, the definition of these types goes beyond semantics. For the more mathematically oriented readers, we provide a mathematical definition of the various terms based on a widely used phenomenological model for survival under drug exposure in Box 1.

Box 1 Mathematical distinctions between antibiotic resistance, tolerance, persistence and heteroresistance Predictive models of the survival of microorganisms exposed to cidal drugs show that measuring the minimum inhibitory concentration (MIC) is not enough to characterize the behaviour, although it is widely used^53,54^. Common phenomenological models for the relationship of the survival, *S*, with the concentration of the drug, *c*, or duration of treatment, *t*, are the Zhi function^55^, Emax or Hill model^56^. In these frameworks, the killing rate, ψ, is described by three main parameters that represent distinct underlying physicochemical mechanisms: the MIC; the minimum duration to kill 99% of the population, MDK_99_; and the Hill coefficient for the steepness of the concentration dependence, *k*.

1 $$S(c,t)={e}^{\psi \cdot t}$$

2 $$\psi \left(c\right)=\frac{{\rm{ln}}(0.01)}{MD{K}_{99}}\cdot \frac{1-{\left(\frac{c}{MIC}\right)}^{k}}{\frac{{\rm{ln}}(0.01)}{{\psi }_{{\rm{m}}{\rm{a}}{\rm{x}}}\times MD{K}_{99}}-{\left(\frac{c}{MIC}\right)}^{k}}$$

This general function predicts how the concentration of the antibiotic and its duration will affect the growth or death of a strain with growth rate without antibiotic, ψ_max_. Note that the common notation of the model uses the following:

3 $${\psi }_{{\rm{m}}{\rm{i}}{\rm{n}}}=\frac{{\rm{ln}}(0.01)}{MD{K}_{99}}$$

In the framework of this model, resistance is defined as an increase in the MIC, whereas tolerance is defined as an increase in the MDK_99_. Thus far, the parameters describe a uniform population. When the population is heterogeneous, it means that at least one of the parameters is heterogeneous.

Heteroresistance entails that subpopulations of cells have a higher MIC than the majority of the population. In typical reports of heteroresistance, it is also assumed that the heritability of the increased MIC is long enough to create detectable colonies^57^.

Antibiotic persistence (which in this context could have been called heterotolerance) entails that a subpopulation of cells have a higher MDK_99_ than the majority of the population. If we assume that the fraction of persisters is α, the survival can be written as the sum of the survival of two subpopulations with different killing rates:

4 $$S\left(c,t\right)=\left(1-\alpha \right){e}^{\psi \cdot t}+\alpha {e}^{{\psi }^{* }\cdot t}$$

The killing rate of the normal population is ψ, as in equation 1, and the killing rate of the persisters is ψ* with a longer MDK_99_.

### Persistent infection versus antibiotic persistence

**Fig. 1:**
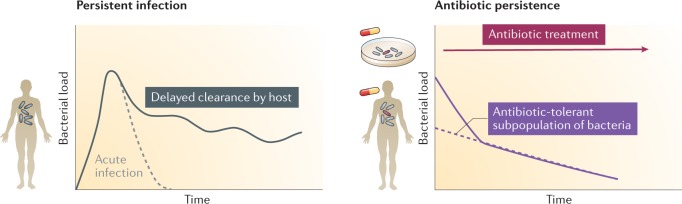
Persistent infections versus antibiotic persistence. Persistent infection is a general term to describe infections that are not efficiently cleared by the host, in contrast to the characteristic acute response that leads to clearance of many pathogens. Antibiotic persistence specifically describes the heterogeneous response of bacterial populations, in vitro or in the host, that results in a delayed clearance of the bacterial load by antibiotics.

First, we would like to distinguish ‘antibiotic persistence’ from ‘persistent infection’^4,5^ (Fig. 1). The latter is generally used to describe infections in the host that are not cleared by the host immune system, whereas antibiotic persistence describes a bacterial population that is refractory to antibiotic treatments, whether in vitro or possibly in the host. Persistent infections are typically multifactorial and involve mechanisms evolved by different pathogens to evade the immune system, such as antigenic mimicry in *Helicobacter pylori*, antigenic variation in *Neisseria gonorrhoeae* and inhibition of phagocytosis^4^ and immune evasion in *Mycobacterium tuberculosis*^6^. As antibiotic persistence specifically addresses the ability of bacteria to survive antibiotic treatments, it may be an additional factor for the prolongation of persistent infections despite antibiotic treatment, for example, in recurrent urinary tract infections^7,8^. Moreover, the same mechanisms may be involved in both immune evasion and antibiotic persistence, for example, biofilm formation^9^.

Historically, the term ‘persistent infection’ was used before antibiotics were available to treat infections. To avoid ambiguity, we suggest using the term antibiotic persistence to distinguish between these phenomena when first mentioned in a publication. The focus in this article is on antibiotic persistence, although for simplicity, tradition and brevity, below, we use the word persistence.

### Persistence versus resistance

**Fig. 2:**
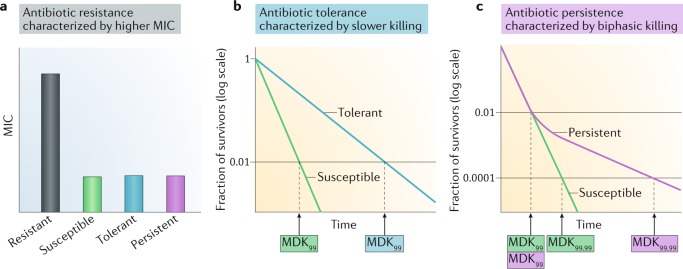
Antibiotic resistance, tolerance and persistence. Resistance, tolerance and persistence are distinct responses to antibiotic treatment that lead to increased survival compared with susceptible cells. **a** | To inhibit the growth of resistant bacteria, a substantially higher minimum inhibitory concentration (MIC) of the antibiotic is needed than for susceptible bacteria. Notably, persistence and tolerance do not lead to an increase in the MIC compared with susceptible bacteria. **b** | By contrast, tolerance increases the minimum duration for killing (MDK; for example, for 99% of bacterial cells in the population (MDK_99_)) compared with susceptible bacteria. **c** | Persistence leads to a similar MIC and a similar initial killing of the bacterial population compared with susceptible bacteria; however, the MDK for 99.99% of bacterial cells in the population (MDK_99.99_) can be substantially higher owing to the survival of the persister cells. Note that pure exponential killing of the susceptible strain is rarely observed because most bacterial cultures have some level of persistence. The data shown are only illustrations and not actual measurements. Parts **b** and **c** are adapted with permission from ref.^3^, Springer Nature Limited (this material is excluded from the CC-BY-4.0 license).

‘Resistance’ is the ability of bacteria to replicate and not just survive in the presence of a drug (Box 2). The most common measure of the level of resistance is the minimum inhibitory concentration (MIC), which is the lowest concentration of the antibiotic required to prevent the replication of the bacteria. A higher MIC corresponds with a higher level of resistance (Fig. 2a). Resistance is inherited and may be acquired by horizontal gene transfer of resistance-encoding genes (for example, encoding antibiotic inactivating enzymes^10^ or efflux pumps^11^) or mutations (for example, leading to modification of the antibiotic target) that confer the resistance phenotype to the bacterial population^12^.

‘Persistence’ is the ability of a subset of the population to survive exposure to a bactericidal drug concentration (Fig. 2). Therefore, persistence is defined only for bactericidal antibiotics. Several features distinguish persistence from resistance. First, the hallmark of antibiotic persistence is the biphasic killing curve (Fig. 2c); that is, the observation that not all bacteria in a clonal culture are killed at the same rate. Second, when persister cells regrow without antibiotics (see below), their progeny give rise to a population that is as susceptible to drugs as the parental population it was isolated from. Third, the level of persistence, namely, the size of the persister subpopulation, will only weakly depend on the concentration of the drug as long as it is far above the MIC. In addition, the survival advantage of persister bacteria is often observed for antibiotic treatments belonging to different classes of antibiotics, for example, β-lactams and fluoroquinolones^13^. Fourth, in contrast to resistant cells, persister bacteria cannot replicate in the presence of the drug any better than the non-persister cells but are killed at a lower rate than the susceptible population from which they arose. This property also distinguishes persistence from heteroresistance, a phenomenon in which a small subpopulation transiently displays a substantially (more than eightfold) higher MIC^14^ (see Box 1).

Box 2 The mechanistic distinction between resistance and tolerance There are numerous mechanisms of antibiotic resistance. The main types of resistance are a reduction in intracellular drug levels (due to reduced uptake or increased efflux), inactivation of the antibiotic or target modification to reduce drug binding. Although mechanisms of resistance are diverse, they typically achieve the same result — reduced antibiotic binding to the target^12^, which allows bacteria to grow. In order to understand tolerance, we need to consider that bactericidal antibiotics kill not by inhibiting the targets, but by corrupting them^58^, leading to toxic products^42,59^. By slowing down these processes in persister bacteria, the activity of targets is diminished, leading to higher survival.

### Persistence and tolerance

‘Tolerance’ and persistence are similar phenomena of increased survival in the presence of an antibiotic without an increase in the MIC. In studies that focus on only a qualitative understanding of the molecular mechanisms, the two terms are often interchangeable^15^. However, persistence has the added attribute of affecting only a subpopulation of cells, whereas tolerance is the general ability of a population to survive longer treatments, for example, by having a lower killing rate (see Fig. 2b), but without a change in the MIC^16^. Persister cells are simply a subpopulation of tolerant bacteria, and persistence could also be called ‘heterotolerance’. Tolerant populations survive the period of antibiotic treatment better, with, typically, a weak dependence on the antibiotic concentration. Therefore, the MIC of tolerant cells is unchanged compared with non-tolerant strains. What characterizes their slower killing, even at high concentrations of the drug, is the time required to kill a large fraction of the population, for example, the MDK_99_, which is the minimum duration of treatment that kills 99% of the bacterial population. Persistence is a special case of tolerance in which a subpopulation of persister cells can survive the antibiotic treatment much better than the majority of the population, as reflected in the biphasic killing curve. Not surprisingly, mechanisms linked to tolerance, such as dormancy (see definitions in Box 3), reduced metabolism and ATP levels, have also been identified in persistence^9^. Therefore, when studying persistence, two mechanisms are of interest, and the first one overlaps with tolerance research whereas the second is specific to persistence: (1) the molecular mechanism of tolerance that enables the persister bacteria to survive, for example, a reduction in their metabolism, and (2) the mechanism that generates heterogeneity in the population^17^, for example, nonlinear mechanisms leading to bimodality by amplifying stochasticity^18,19^. Finally, several persister subpopulations may coexist; therefore, a multimodal killing curve may occur.

Box 3 Definitions **Antibiotic resistant cell**

An antibiotic resistant cell is a cell that survives antibiotic treatment by carrying a resistance factor (for example, an efflux pump). Resistance factors enable resistant bacteria to grow at antibiotic concentrations that would prevent the growth of more susceptible bacteria.

**Antibiotic tolerant cell**

An antibiotic tolerant cell is a cell that survives treatment with an antibiotic, without carrying a resistance factor, and that can regrow after removal of the antibiotic. Often, tolerant cells are non-growing before antibiotic exposure, but not necessarily. Tolerance factors enable bacteria to survive the duration of treatment that would kill more susceptible bacteria. These tolerance factors can be environmental or genetic.

**Antibiotic persistence**

Antibiotic persistence is a population-level phenomenon that historically has been derived from the observation of biphasic killing curves, indicating the presence of two subpopulations, consisting of cells that are killed fast by the antibiotic and tolerant cells that may survive. By definition, the term antibiotic persistence is always connected with a heterogeneous population, in which only a part of the population consists of tolerant cells.

**Tolerance**

Tolerance is a population-level phenomenon that enables the population to survive the duration of a transient antibiotic treatment several times above the minimum inhibitory concentration (MIC) without a resistance mechanism.

**Persister cell**

A persister cell is a tolerant cell originating from a population that displays antibiotic persistence.

**Dormancy**

Dormancy reflects the state of a bacterium that does not grow and has decreased activity when compared with growing cells or even typical stationary phase cells. This term is often also used for single cells that are viable but do not grow despite environmental conditions that support growth. Dormant bacteria are often tolerant to many antibiotics because of their growth arrest or their decreased metabolism^60^. However, tolerance and persistence may arise without dormancy.

### Single-cell versus population phenotype

Because the definition of antibiotic persistence is anchored in the heterogeneity of the response to antibiotics in the population, it is a population-level phenotype. However, tolerance can be the attribute of a whole population that is killed at a slow rate as well as of a single cell that manages to survive an extensive treatment (see definitions in Box 3).

Genetic mutations can increase the tolerance of a strain if they result in slower killing. Similarly, genetic mutations can increase the persistence of a strain either by reducing the killing rate of the persistent subpopulation even more or by increasing the fraction of that subpopulation, as, for example, in the *hipA7* high persistence mutant^20^. The population level of high persistence is then genetically inherited.

## Types of persister bacteria

Whether a single general or multiple specific molecular mechanisms underlie persistence is still under debate^21–23^ and therefore will not be discussed in this article. However, distinct ways for generating persister bacteria in a culture have been identified. Distinguishing between the types of persistence identified thus far is crucial because each type requires a different procedure to measure the persistence level.

### Triggered persistence

**Fig. 3:**
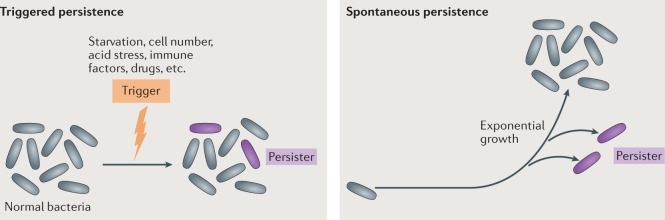
Triggered versus spontaneous persistence. Triggered persistence requires a trigger for bacteria to become persisters (left). The persistence level will then depend on the intensity and duration of the trigger. For example, a common trigger for persistence is starvation. Even when the trigger is removed, persister bacteria may retain their phenotype for an extended duration. Spontaneous persistence occurs when the bacteria are in steady-state exponential growth (right). A fraction of the population switches stochastically to the persister phenotype at a rate that is constant during growth. Such steady-state conditions can be found in chemostats or serially diluted cultures, and care must be taken to ensure that the persisters do not originate from the inoculum or from the culture being too close to entry into the stationary phase.

In most observations of persistence described to date, the fraction of persister bacteria is generated upon a stress signal, the most common one being starvation (Fig. 3). This type of persistence, here termed triggered persistence, was previously called type I persistence^24^. Even when the signal is removed, for example, by diluting a starved overnight culture in fresh medium, persister cells may still linger for extensive periods and be the ones found in the surviving fraction. Even when the culture is allowed to resume growth for a few hours and to reach what seems to be ‘exponential growth’, a fraction of the persisters triggered by the previous starvation may remain in a lag phase. Therefore, the lag time distribution of single cells after starvation or exposure to a stress is an important factor to take into account as it may determine the persistence level^25,26^.

Many stress conditions have been shown to generate triggered persistence, including limitation of different nutrients^27^, high cell number^28^, acid stress, immune factors^29^ and exposure to immune cells^30^.

Confounding results can occur when the antibiotic itself serves as a trigger for growth arrest, causing drug-induced persistence^31^ and sometimes paradoxical lower killing at high drug concentration^32^. In this case, instead of killing the cells, a bactericidal antibiotic becomes bacteriostatic for a subpopulation of cells that respond to the antibiotic signal itself, for example, by activating a stress response that enables them to survive^31,33^. This type of persistence does not depend on the history of the culture before exposure to the drug^34^ and therefore may be attributed to spontaneous persistence. However, because in this case persistence relies on the response of the cells to the antibiotic, it may be more specific to the class of antibiotic used and its concentration than other forms of persistence.

### Spontaneous persistence

Persistence may be observed without any trigger and when the culture is in steady-state exponential growth and all parameters are kept constant, that is, during balanced growth (Fig. 3). In this case, persisters may occur spontaneously, and their fraction remains constant as long as the steady-state growth is maintained. Spontaneous persistence was previously called type II persistence^24^. This form of persistence seems to be much less common than triggered persistence.

### Effect of mutations

Importantly, apart from the environmental triggers mentioned above, all types of persistence may be increased (or reduced) by mutations. Although within a clonal population, persister and non-persister bacteria are typically isogenic, mutations have been identified that are able to increase the level of persistence or tolerance. For example, one of the first identified high persistence mutations, the *hipA7* mutation^20^, increases the level of triggered persistence in *Escherichia coli* by orders of magnitude, reaching persistence levels of about 20% of the population, whereas it is typically below 0.1% for wild-type strains. A high persistence mutation can be viewed as a tolerance mutation with partial penetrance in the whole population; only the subpopulation of persister bacteria will exhibit a phenotype owing to the mutation and will die slower. A high tolerance mutation reduces the killing rate of the whole population. In other words, 100% persistence is equivalent to tolerance.

We stress the fact that the definition of persistence presented here is not directly linked to a specific mechanism or to a physiological state of the bacteria. Rather, it is defined by the time-kill assay; therefore, we outline below some important considerations for increasing the reproducibility and reliability of persistence detection. Typical microbiology procedures developed for measuring uniform bulk phenomena need to be carefully re-evaluated when measuring survival that is dominated by a small subpopulation of cells.

Although we focus here on antibiotic persistence of bacteria, we believe that the definitions and guidelines should be relevant for heterogeneous responses to other drugs such as antifungals^35^ and anticancer treatments^36^.

## A guide to measuring persistence

The starting point for identifying persistence is the time-kill assay, which measures survival of bacteria at different time points during exposure to the antibiotic. Survival is defined as the ability to regrow when the antibiotic is removed. The hallmark of persistence is the bimodal (or multimodal) killing curve (Fig. 1). However, observing bimodal killing alone is not enough, and several additional steps are required to evaluate whether the bimodality results from persistence or from resistance and to differentiate between the different types of persistence mentioned above. A standardization of the assays and a clear description of the conditions used are required to enable comparing different strains or conditions and results from different laboratories.

### Does the bimodal killing curve really reflect persistence?

First, a bimodal killing curve may be due to resistant mutants. To rule out this effect, surviving bacteria that are clearly in the tail of the survival curve should be regrown in the same conditions and exposed again to the same antibiotic treatment. Persistence requires that the killing curve remains the same as in the initial inoculation^37^. If resistant mutants were responsible for the slower killing rate in the first killing curve, the second assay will show reduced killing of a much higher proportion of the population than in the first assay.

Second, in order to distinguish persistence from transient modes of resistance such as heteroresistance^14^, the killing curve should be performed at high antibiotic concentration, at least several times the MIC, to efficiently kill bacteria that have a higher MIC than the rest of the population. The killing rate should only weakly depend on the antibiotic concentration. Strong dependence on the antibiotic concentration (scaled with MIC) reflects phenomena linked to resistance.

Third, experimental pitfalls that may result in bimodal killing should be ruled out. One of the most common reasons for a decrease in the killing rate is degradation of the drug with time. Therefore, it is important to test that the killing efficacy of the drug itself does not decrease with time because of natural degradation of the drug, uptake by bacteria or changes in the medium. Another reason for survival of some bacteria may be their adhesion to the walls of the culture vessel^38^, where the antibiotic may not efficiently kill them.

Finally, in addition to clearly stating in which conditions the time-kill assay is performed, care should be given to the recovery conditions, when bacteria are allowed to grow after removal of the antibiotic. The precise conditions for the evaluation of survival after treatment should be described, such as the washing out of the antibiotics, the medium in which the bacteria are recovered and the time that has passed from the exposure to the antibiotics until the exposure to the recovery conditions. For example, it has been shown that keeping the bacteria in non-growing conditions after treatment may increase their survival^39^. In addition, bacteria recovering from an antibiotic treatment may have a delayed regrowth either because they are in the tail of the lag time distribution^26,40^ or because of the post-antibiotic effect^41,42^, which results in the delayed growth of bacteria after treatment. Therefore, evaluating the survival by counting colonies should be done not only after the typical appearance time of colonies but also several days later.

### Measuring triggered persistence

As the trigger is an integral part of triggered persistence, the trigger duration, intensity and exact conditions should be clearly mentioned and kept the same between experiments. For example, one of the most common triggers of persistence is starvation. Many reports used an ‘overnight culture’ as inoculum. This overnight culture has been exposed to several stress signals during starvation, such as high cell density, stringent response and altered pH, that may trigger persistence, and therefore inevitably still contains bacteria that experienced a trigger. In this example of triggered persistence, the persistence level will depend strongly on several parameters, including the size of the inoculum, the time that has elapsed since the inoculum was regrown and the duration of starvation during the previous overnight culture^26,40^. Typically, to obtain reproducible results, the time between the trigger for persistence and the exposure to antibiotics should be minimized to avoid the uncontrolled loss of persister bacteria that switch back to normal cells.

### Measuring drug-induced persistence

The conditions for measuring drug-induced persistence are the same as for measuring spontaneous persistence, namely, steady-state growth, as the trigger is the drug itself and should be applied in steady-state conditions to avoid stationary-phase-induced persistence. Without further characterizations, the spontaneous and drug-induced persistence are difficult to distinguish. In this case, direct observation of single cells as they respond to the antibiotics is needed^43,44^, or a dissection of the molecular mechanism that allows the bacteria to respond to the drug by activating a stress response^31^. Earlier attempts to characterize drug-induced tolerance or drug-induced persistence made use of the minimum bactericidal concentration (MBC)^38^. The MBC is the concentration required to kill bacteria. Some drugs may arrest the growth at the MIC but require a higher concentration to kill. If the drug itself induces persistence at the MIC, a higher concentration may be required to reach killing. Drug-induced persister bacteria have a higher MBC than the rest of the population, but their MIC is unchanged.

### Measuring spontaneous persistence

In contrast to triggered persistence, which is determined by the history of the culture, the rare spontaneous persistence should be measured in conditions of steady-state (also called balanced) growth so as to avoid the effect of the past growth conditions. This measurement can be achieved in a chemostat or by subdiluting the culture several times^37^ before performing the time-kill assay, making sure to dilute the inoculum to below the persistence level^24^. As spontaneous persistence is a steady-state phenomenon, care should be taken to evaluate whether the culture remains in steady-state growth, also after the inoculum influence has been ruled out. For example, a common pitfall is to perform the time-kill assay without diluting and subculturing the bacteria for enough time to eliminate the persister bacteria triggered by past stationary phase growth. Another common pitfall is to perform the time-kill assay when the culture is too close to the next stationary phase, which again may trigger the formation of persister cells. Even if the culture seems to be growing exponentially, it may no longer be in balanced growth and persister formation may be already triggered at a cell density that is ten times lower than the maximal density^45^. The spontaneous persistence fraction should remain constant with time in steady-state growth conditions. A simple way to test that the results do not depend on the cell density is to perform the same experiment at a twofold lower density and verify that the persistence fraction remains the same.

### Regrowth of persister bacteria

An inherent part of the persistence phenomenon is the ability of persisters to eventually resume growth. As evidenced by the low killing rate displayed in the second phase of biphasic killing curves (Fig. 2c), persisters may resume growth at a low and constant rate, independently of the presence of the drug. Only persisters resuming growth after cessation of the antibiotic treatment will give rise to a new population of susceptible bacteria.

Single-cell observation often shows non-growing cells that remain intact during exposure to the drug. However, regrowth must be documented^30^ to illustrate that bacteria have survived exposure to the drug before those can be dubbed persisters.

## Conclusion

There has been a sharp increase in the interest for antibiotic persistence in the past years in the background of growing concerns about antimicrobial resistance. The observation that triggered persistence evolves fast in vitro^46,47^ and can be followed by the evolution of resistance^48^ suggests that persistence may be evolving quickly in the host as well. It has been suggested that the presence of antibiotic persister cells is responsible, at least partly, for lack of clearance of pathogenic bacteria by antibiotic treatment. Indeed, even in the absence of any antibiotic resistance, many bacterial infections are hard to treat and tend to relapse (such as tuberculosis, lung infections in people with cystic fibrosis, systemic infections with *Salmonella*, tonsillitis and urinary tract infections). The underlying reasons are most likely multifactorial, with suboptimal pharmacodynamics in the host probably playing a major role in some instances. However, it is also clear that non-growing bacteria^29^ and high-persister-forming mutants are selected over time in patients exposed to repeated doses of antibiotics^49,50^. Further work is needed to evaluate the possible impact of persister cells on the treatment outcome of bacterial infections and to find ways to fight them^15^. As seen above, many pitfalls exist even for in vitro analysis of persistence, and controlling the experimental conditions is crucial. The understanding of persistence in the host, in which our knowledge of the conditions is scarce, is orders of magnitude more challenging^51,52^. It is our hope that the guidelines outlined in this article will enable a consensus on in vitro measurements and pave the way for designing protocols adapted to the clinical evaluation of antibiotic persistence.
